# Myoclonic Jerks and Ataxia: A Case of Rare Neurological Side Effects of Amiodarone

**DOI:** 10.7759/cureus.28412

**Published:** 2022-08-25

**Authors:** Vanessa Milan-Ortiz, Anirudh R Damughatla, Saivaishnavi Kamatham, Zain N Jawad, Shakeela Shakoor

**Affiliations:** 1 Internal Medicine, Wayne State University Detroit Medical Center, Detroit, USA; 2 Internal Medicine, Wayne State University School of Medicine, Detroit, USA; 3 Internal Medicine, Veterans Affairs Medical Center, Detroit, USA

**Keywords:** amiodarone in the elderly population, amiodarone, neurological side effects of amiodarone, ataxia, myoclonic jerks

## Abstract

We present the case of an 85-year-old man with a known medical history of recurrent ventricular arrhythmia and paroxysmal atrial fibrillation who was started on an amiodarone loading dose four days before presenting with tremors and balance impairment. Amiodarone's adverse effects in various systems have been well studied, although this is not the case with neurotoxic side effects. In this article, we discuss the neurotoxic side effects caused by amiodarone and the importance of a complete physical examination when starting this medication for supraventricular and ventricular arrhythmias.

## Introduction

Amiodarone is a class III antiarrhythmic agent commonly used to manage supraventricular and ventricular arrhythmias. Amiodarone is widely known for its toxic effects on multiple organ systems - eyes, thyroid, lung, skin, and liver [1]. The adverse effects of amiodarone in those systems have been reported more frequently; however, this is not the case with neurotoxic complications. Tremors, ataxia, and peripheral neuropathy are the most common neurological adverse effects. Although, cognitive impairment, encephalopathy, parkinsonism, optic neuropathy, myoclonus, and myopathy have also been reported [2]. Some of the adverse effects of amiodarone are related to the dose and treatment duration [3]. However, the literature does not specify how early in the treatment course the neurological effects can be seen. We present a case of an elderly gentleman with acute onset ataxia, severe muscle cramps, and upper and lower extremities tremors at rest and on exertion after receiving one dose of intravenous (IV) amiodarone and three days of the loading dose (400 mg twice daily {BID}) of oral (PO) amiodarone.

## Case presentation

An 85-year-old male with a history of recurrent ventricular tachycardia status post implantable cardioverter-defibrillator (ICD), paroxysmal atrial fibrillation status post direct cardioversion and ablation, heart failure with reduced ejection fraction (left ventricular ejection fraction {LVEF} 20%), hypertension, and hyperlipidemia presented with sudden uncontrollable upper and lower extremity tremors and muscle cramps that started three days after initiating amiodarone PO loading dose. The patient was treated for ventricular tachycardia with procainamide during his last admission one week before our evaluation. The patient was also administered one IV amiodarone dose and discharged on amiodarone 400 mg PO BID. The patient was given the following instruction to taper down amiodarone dose: 400 mg BID for one week, followed by 200 mg BID for two weeks, and then transition to 200 mg daily. Patient long-term medications included pravastatin 20 mg nightly, aspirin 81 mg daily, apixaban 2.5 mg BID, lisinopril 10 mg daily, metoprolol succinate 50 mg daily, furosemide 20 mg daily when needed, spironolactone 12.5 mg daily, and pantoprazole 40 mg daily.

On presentation, the patient had involuntary generalized muscle jerks at rest, more prominent in the lower extremity, ataxia with dysmetria, and intact motor and sensory function without cranial nerve palsy. Finger-to-nose and heel-to-shin testing were positive, while the Romberg test was negative. Upper and lower extremities tremors worsened when reaching a target. Ophthalmology eye examination demonstrated intact extraocular movements, and fundoscopic evaluation was stable compared to previous evaluations. Laboratory studies showed normal thyroid-stimulating hormone (TSH), glucose, ammonia, creatine phosphokinase (CPK) levels, and baseline kidney function (Table 1).

**Table 1:** Pertinent lab results at the time of admission. TSH: thyroid-stimulating hormone; BUN: blood urea nitrogen

Lab tests	Values	Normal range
Ammonia	14 umol/L	11-60 umol/L
TSH	1.91 uIU/mL	0.27-4.20 uIU/mL
Folate serum	19.6 ng/mL	≥4.5 ng/mL
Vitamin B12	673 pg/mL	232-1245 pg/mL
Glucose	115 mg/dL	74-109 mg/dL
Sodium	141 mmol/L	136-145 mmol/L
Potassium	3.8 mmol/L	3.5-5.1 mmol/L
Magnesium	2.1 mg/dL	1.6-2.4 mg/dL
Phosphorous	2.6 mg/dL	2.4-2.1 mg/dL
BUN	16 mg/dL	6-20 mg/dL
Creatinine	1.1 mg/dL	0.7-1.3 mg/dL

**Figure 1:**
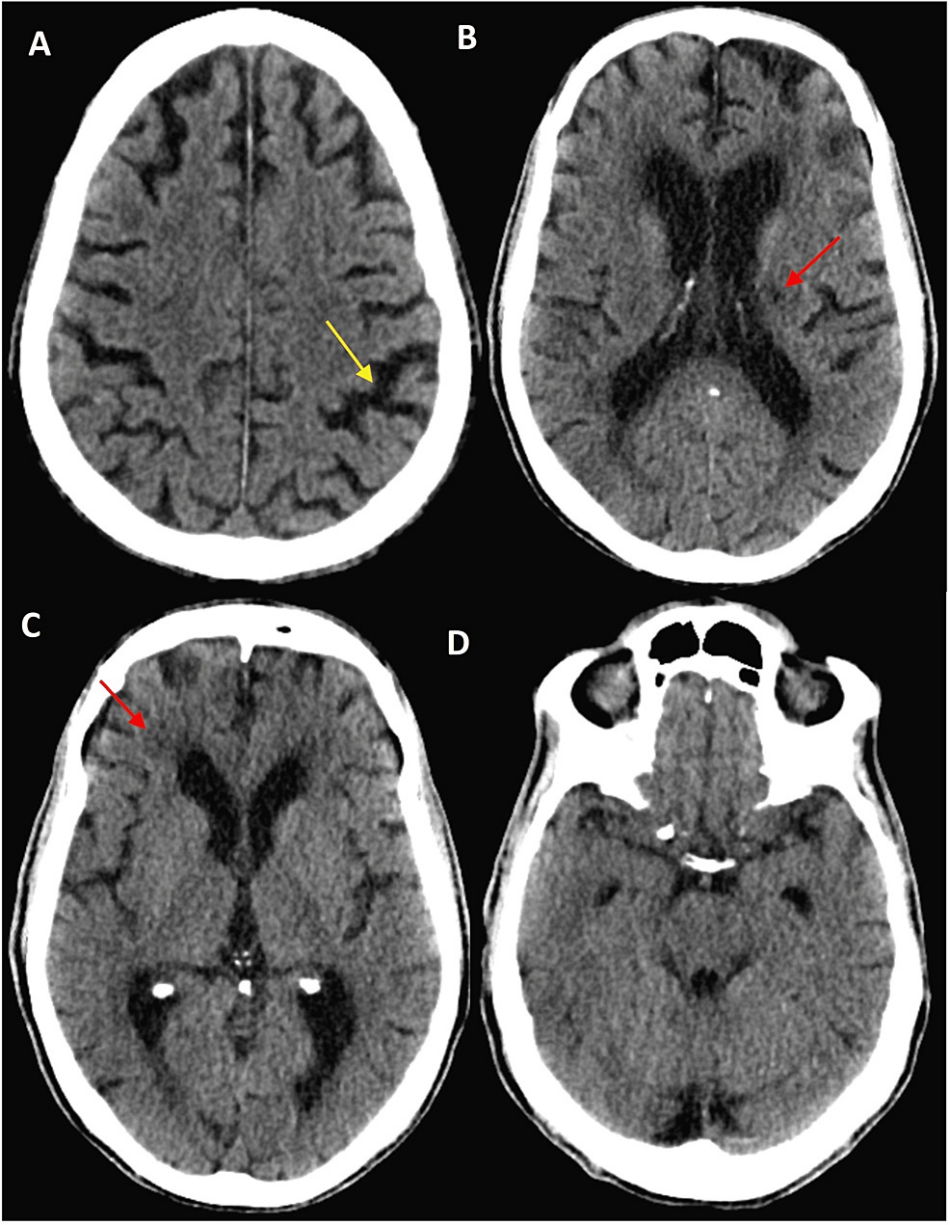
CT head without contrast of the patient. The images show (A) parenchymal volume loss (yellow arrow) and (B and C) chronic white matter microvascular ischemic disease (red arrows); (D) the posterior cranial fossa and anatomy are essentially normal for the patient's age.

The head CT scan was significant for parenchymal volume loss with a chronic microvascular ischemic disease; however, there were no acute intracranial hemorrhage or ischemic processes (Figures 1A-1D). Brain MRI and magnetic resonance angiography (MRA) would be the preferred imaging modality to evaluate for brain structural and vascular abnormalities; however, the brain MRI could not be performed as ICD was incompatible with our hospital MRI machine. The dilemma was decreasing the antiarrhythmic dose versus stopping it, but the patient had a significant history of recurrent VT refractory despite undergoing cardiac ablation. The cardiology team also evaluated the patient during this admission and agreed with accelerated tapering of the amiodarone. On day two of admission, amiodarone dose was decreased to 400 mg daily, and on day four, it was reduced to 200 mg daily. After one week of decreasing the dose, the patient's tremors significantly improved. The patient was discharged to a physical rehabilitation center on amiodarone 200 mg daily. He was followed up again in a few weeks at the rehabilitation center, where the patient noted that his tremors were minimal and completely resolved.

## Discussion

Amiodarone has been routinely used in the past as a first-line for the management of supraventricular and ventricular arrhythmia. However, it has become a second-line agent due to the development of other alternative treatments, such as cardiac ablation and implantable cardioverter-defibrillator [4]. Amiodarone's pulmonary, thyroid, and dermatological side effects have been widely studied; nonetheless, this has not been the case with neurological side effects. Amiodarone can cause a broad spectrum of neurological manifestations, including tremors, gait ataxia, cognitive impairment, peripheral neuropathy, myoclonus, and rarely, parkinsonism and myopathy [2], with incidence of these adverse effects reported to be ranging from 2.8% to 74% [5]. Amiodarone has recommended oral loading dose is 400 mg to 600 mg daily in divided doses for two to four weeks, followed by a maintenance dose of 100 mg to 200 mg daily. The loading dose cannot exceed 2.4 g in 24 hours. Van Herendael and Dorian demonstrated a higher occurrence of neurological and gastroenterological adverse effects when amiodarone plasma levels are >2.5 mg/L [6]. The mechanism of central neurotoxicity is unknown, but it is believed that amiodarone and mono-N-desethylamiodarone (amiodarone major oxidative metabolite) can inhibit liposomal phospholipase, leading to phospholipid accumulation in tissue [5,7]. The neurological adverse effects can develop at any time during treatment. Although, Orr et al.'s study demonstrated that the length of time receiving therapy was the leading risk factor for developing amiodarone neurotoxic effects [2].

Raeder et al. reported that neurotoxic side effects could be seen 40 days to 15 months after initiation of therapy [8]. At the same time, other studies have shown that these effects can be seen as early as three months after starting amiodarone [8]. Vorperian et al. meta-analysis concluded that exposure to low doses of amiodarone for 12 months doubled the odds of developing neurologic adverse effects compared to placebo [9].

In our case, the patient presented to the emergency department with myoclonic jerks and ataxia a few days after starting amiodarone. Other causes of myoclonic jerks and ataxia were evaluated. The patient was found to have normal thyroid function, liver function, CPK, glucose, ammonia, baseline kidney function, and CT scan without any acute abnormalities and only chronic parenchymal volume loss with a chronic microvascular ischemic disease. In addition, the patient was not on any other medication known to cause acute myoclonic jerks and ataxia; moreover, he has been taking them for more than a year. Other etiologies such as liver failure, renal failure, and infection have been ruled out. Once the amiodarone dose was reduced, as it was the only possible etiologic agent, the patient's symptoms started to resolve. Over a few weeks, the patient's symptoms significantly improved. When calculated, the patient had a Naranjo score of 5, which suggests that the patient's myoclonic jerks and ataxia are probably due to the high dose of amiodarone [10].

This case shows us that it is essential to do a thorough neurological examination when initiating amiodarone therapy and then periodically evaluate for the development of adverse effects. Neurological adverse effects can subside or diminish after discontinuing or adjusting amiodarone dosage [6]. Although amiodarone is highly lipophilic, its plasma concentration decreases around 25% after stopping the medication for a few days [4]. Krauser et al. reported that neurological disturbances improved or resolved after days of ceasing the medication, while in a few other cases, it has taken weeks to months [8].

The literature demonstrates that levetiracetam has been used effectively in decreasing myoclonus jerks. Deik and Shanker presented a case of a 90-year-old man who developed action-induced myoclonus jerks one year after initiating amiodarone therapy. At the same time, Celli et al. reported a case of a 60-year-old man who also presented with myoclonus jerks three days after reinitiating amiodarone. In both cases, the patients were administered levetiracetam to improve their quality of life by reducing myoclonic jerks [11,12]. Levetiracetam is an antiepileptic drug that modulates the release of neurotransmitters by binding to the synaptic vesicle protein SV2A in the brain. It is the first line for treating reflex cortical myoclonus by increasing the inhibitory processes within the sensorimotor cortex [12].

## Conclusions

The case highlights the importance of a thorough physical and laboratory evaluation at baseline and periodic assessment for patients initiated on amiodarone therapy. It is also imperative for physicians to consider that medication side effects can present typically or atypically in the geriatric population, and rare adverse effects can be more prevalent due to the pharmacodynamics and pharmacokinetics of an aging body. Maybe a smaller dose of amiodarone should be considered in the elderly population for both loading and maintenance doses.
